# Designing Online Courses: 12 Tips for Health Professions Educators

**DOI:** 10.15694/mep.2020.000117.1

**Published:** 2020-06-01

**Authors:** Elisabeth Schlegel

**Affiliations:** 1 Donald and Barbara Zucker School of Medicine at Hofstra/Northwell

**Keywords:** Online Course, Instructional Design, Curriculum Planning, Health Professions Education.

## Abstract

This article was migrated. The article was marked as recommended.

With the changes brought on by the public health concerns during the spring of 2020, face-to-face teaching was replaced by remote teaching in a rapid fashion. Learning management systems (LMSs) and video conferencing services were rapidly utilized to deliver online learning of previously in-person sessions. As the familiarity with remote teaching increases, designs of previously taught face-to-face courses will be revisited. Online teaching and learning provide great options for self-paced education in the dynamic environment of health professions education. Creative use of tools and collaborative web-based technology enables learner-centered pedagogy and the establishment of a community of learners. We offer practical tips for designing and implementing a generalized online course for health professions educators that appeal to diverse learners through meaningful use of different types of media.

## Introduction

With the changes brought on by the public health concerns during the spring of 2020, face-to-face teaching was replaced by remote teaching in a rapid fashion. Learning management systems (LMSs) and video conferencing services were rapidly utilized to deliver online learning of previously in-person sessions. Little time was left to redesign and review courses for optimizing learning or to realign objectives with existing instructional materials and assessments. At the same time, assessing and improving the technical use of online learning environments across the globe led to rapid adoption of new learning and teaching procedures and provided learning and insights about the use of technology for teaching.

As the familiarity with remote teaching increases, time will become available to revisit designs of previously taught face-to-face courses. With the hope of returning to genuine human contact and in-person teaching, advantages of remote teaching - such as ease of planning and clarity of expectations for both teacher and learner - can be applicable in the hectic healthcare education environment. While synchronous teaching is currently preferred to continue programs throughout the year, asynchronous teaching and learning provide great options for additional education that accommodates the pace and learning preference of the learner. However, meaningful planning and effective course design is necessary to ensure a smooth educational landscape that supports the learner. Prudent use of educational tools, e.g., discussion board or breakout rooms, ensures learner-centered education and supports proper course design if aligned with objectives, content, and assessments.

We offer practical tips for designing and implementing a generalized online course for health professions educators. We aim to provide a straightforward roadmap for course planners that can be accomplished in a step-by-step fashion.

## Tip 1: Establish Your Course Design and Flow

The online learning environment becomes the instructor-learner interface and educational landscape for learners. With human contact momentary suspended, prudent design and appropriate use of technology can introduce a great learning experience conducive to learning and growth.

Mostly due to learning management systems hosting online courses, the organization into a sequence of subject matter delivered over time becomes more obvious compared to in-person taught courses. Comparable to face-to-face programs, effective course design ensures student learning, ease of facilitation, and overall alignment of course components. With clear organization of course topics delivered over time, planning of learning becomes facile for students and instructors. With a minor trade-off in flexibility of content, one great advantage of proper course design is clear expectations for both facilitator and learner.

Many curricula in health professions education are developed following the sequence of David Kern’s six-step approach to curriculum development, starting with (1) a Problem Identification/General Needs Assessment, followed by the (2) Targeted Needs Assessment, establishing of (3) Learning Objectives, (4) Educational Strategies, (5) Implementation, and (6) Evaluation and Feedback ( Thomas *et al*., 2016). The importance of these steps still prevails; however, the sequence of the planning process changes. For online course design, a best practice approach is to identify knowledge, skills, and attitudes obtained by completion of the course first (Vai and Sosulski, 2015).

By determining desired results and goals first - as expressed in learner-centered course outcomes - additional components such as assessments or activities can be aligned and developed with instructional technologies available ( McTighe and Wiggins, 1999). This will also provide a feasibility check and a grasp of the time resources needed to complete the course design and development.

Table 1 shows a schematic of an online course with three learning modules. The components are explained as follows.

**Table 1.** A schematic of an online course with three learning modules

Online Course Topic	
**Announcements**	
**Introduction to Course Content**	
**Module 1**	SMART Learning Objectives 1
	Content 1
	Activities 1
	Assessments 1
**Module 2**	SMART Learning Objectives 2
	Content 2
	Activities 2
	Assessments 2
**Module 3**	SMART Learning Objectives 3
	Content 3
	Activities 3
	Assessments 3
**Course Outcomes**	
**Evaluation**	

## Tip 2: Determine the Learning Modules or Learning Units

Next, the content needs to be broken down into manageable portions, termed learning modules or learning units. Learning modules are tied to dates of completion and may encompass different time frames, such as hours or individual weeks. The module duration should be consistent throughout the course. Learning management systems offer easy creation of such modules. Teachers should note that this organization of content can also be reflected in the course syllabus.

Each module requires SMART learning objectives - what students are expected to master by the completion of the module - which will be communicated to the learner. Each modular SMART learning objective needs to be aligned with and reflect the overarching course outcomes, which the student will have mastered by the end of the course.

When establishing modules online, it may be easy at first to start with one and create a set of similar modules for the remaining weeks or days. While developing individual module learning objectives, course designers should make note of the ideas of educational strategies that emerge in their mind. While uniformity of modules sets clear expectations, predictability and repetition can be avoided through different educational strategies and assessments ( Boettcher and Conrad, 2016).

## Tip 3: Determine the Assessments for Each Learning Module or Learning Unit

With the learning objectives established, appropriate assessments can be determined. In a learning management system, options for assessment are wide and varied, but typically fall into two present options, such as tests and assignments. In both cases, the facilitators are expected to provide detailed instructions about expectations.

Automatically graded quizzes and tests help the instructor obtaining feedback about learner mastering of content quickly. However, and comparable to traditional assessments, the type of learning objective determines the type of assessment. As an example, knowledge-based learning objectives may be best assessed through a test, while attitudinal learning objectives may be best assessed through reflective writing.

Both course developers and facilitators must keep in mind that complex assessment types such as course papers, group projects, or forum posting require more time in grading but afford deeper insights about learner progress. To clarify expectations to the learner, rubrics need to be developed and posted.

Depending on the content, one best practice might be scheduling frequent quizzes for self-assessment and as formative feedback at the end of each module, and cumulatively written assessments at the conclusion of the course. Formats of assessments can include movie files or links to online products, e.g., YouTube videos, developed by groups of students. One great feature in the learning management system is the online grade book to communicate grades and feedback to learners. It enables both instructor and learner to review and track academic progress throughout the course (Vai and Sosulski, 2015).

## Tip 4: Determine the Content and its Facilitation

As outlined by Thomas and colleagues, educational strategies include both content and methods. While content refers to the specific material to be presented, methods are the ways in which the content is facilitated. Comparable to face-to-face education, adult learning strategies in online environments enable learner-centered education and establish communities of learners ( Thomas *et al*., 2016). Providing multimedia sources on the LMSs supports tenets of andragogy, e.g., self-discovery or simulated experiences, and enable learners to participate in the learning process ( Knowles, 1984).

In effectively designed online courses, presentation of content is tightly connected with learning activities that engage the learner. LMSs are equipped with features that facilitate learner-centered communication and support text, audio, video, or images and capture interest and inspire imagination of learners. Thus, content can be presented invitingly and barrier-free.

Live video presentations in synchronous learning can include interaction with the audience through text-based chats or live audio. Comparable to face-to-face instruction, Socratic dialogue and open questions support a dynamic class discussion, supported by technology. Hand-raising features indicating readiness to participate live in a discussion are available. This way, a community of learners share multiple viewpoints and critical thinking as a social experience (Vai and Sosulski, 2015).

A great feature of LMSs are breakout rooms. This feature allows to swiftly break the class into groups for a given time to work on a specific topic. Once the time expired the groups return to the main group, ready to report out. As a very useful tool, some LMSs offer whiteboards for the entire class to collaborate and display their report. Traditional flipped classroom design for live sessions can be used throughout the course with the online class time being freed for small groups or active learning exercises ( Moffett, 2015). Facilitators of a vibrant learner-centered online environment might have to allow themselves time to get comfortable with the dynamic in the virtual classroom. If possible, a co-host can monitor the chat and the raised hands and support the facilitator answering questions and discussing with individual students. It is advisable to shorten presentations to make room for student interactions.

## Tip 5: Select Activities and Tools Supporting Collaboration

Varied adaptable activities and tools are available for online courses. However, activities must be aligned with the content, the course outcomes, and the preference of the target audience. Students should be frequently engaged in meaningful activities throughout the course. The course designer will initially plan learner engagement, which is then fine-tuned based on student - and educator - feedback over time.

One of the most well-known tools is the discussion forum (AKA discussion board), which may be used to accompany the class discussion or as a core activity and assessment tool. Three types of discussion forums are distinguished: (1) social; (2) general; and (3) topic driven ( Shaul, 2007, as cited in Vai and Sosulski, 2015). In general, discussions are asynchronously set up as mandatory written exchanges for the entire class and require rubrics for grading. As a means of community building, the entire class, including the teacher, participates in discussion forums. Typically, the course designer or instructor sets up the topic with a title. Learners reply by creating threads. Discussion threads can serve as a social forum for introductions or as interactive class discussions with questions and answers. Nongraded discussion threads set up as “water cooler talk” might allow peer-to-peer discussion that supports problem solving and community building.

A reflective activity oftentimes included in the LMS is blogs, which allow multiple entries useful for journaling activities. Blogs allow posting text, images, links, and audio or video file postings.

Comparable to face-to-face instruction, group projects and team research projects are great collaborative activities that allow developing a course product together. Such products may include documents but also videos that can be uploaded to YouTube and shared with the class. Final products can then be displayed in the discussion forum.

## Tip 6: Try it: Multimedia to Support Learning

Many learners participating in digital courses are accustomed to technology being predominantly visual and auditory. Thus, the course designer must create multimedia-rich environments and interactive tasks, rather than reading and writing. On the other hand, creative acts involving taking photographs, writing, or making music engage individuals in a process of reflection ( Gauntlett, 2007). Digital courses using LMSs can take advantage of a wide range of media either for providing content, assigning learning activities, or assessing the learners’ progress or reflective journey ( Sandars, Murray and Pellow, 2008).

While most meaningful resources in health professions education are text-based, other media are available to captivate the audience and either complement or deliver the primary content. Thus, documents describing a study and available through the LMS are vital, yet can be brought to life through uploading YouTube videos, TED talks, web links, graphs, or images that inspire reflection and discussion. Course designers should check with their librarian to ensure the media file you intend to post can be used for educational purposes, i.e., abides by copyright rules, or to search for openly licensed images (Vai and Sosulski, 2015). In recent years, great auditory media such as podcasts were developed for a wide range of medical education subjects. These, too, allow for enrichment of education and can be assigned either as content or pre-work for flipped classroom-style synchronous sessions.

Finally, course designers must allow themselves time to learn what works best. A novice designer is encouraged to experiment and ask for frequent feedback from, e.g. students testing the course components. Once a course is established, individual content pieces can still be exchanged (with good measure), allowing growth for the students, instructor, and designer.

## Tip 7: Streamline Communication with the Class

Communication with the class or individual students can create momentum and is supported by the LMS. One well-known feature is the announcement function, which allows posting news to the course site as well as sending it to individual students via email. Best practice for using announcements is posting and sending new information prior to the start of a new unit, with a brief review of the content to come. Recurring announcements get the class ready for the next unit, put everybody on the same page, and create teaching presence ( Mealey, 2019).

The instructor might also enact social and managerial roles at the beginning of the course to establish rapport and clarify the intended journey of the class. This may include a comprehensive orientation at the beginning of the course in the announcement. It is recommended to briefly review the overall goals but also the requirements due in the first weeks, even when stated in the course syllabus (Morris and Finnegan, 2008).

Emails can be used to interact with individual students and groups of students. In order to avoid answering frequent questions to individuals only, a discussion forum can be established with questions posed to the instructor.

## Tip 8: Strive for a Clear Course Layout

Clear, attractive page design and layout promotes communication and ease of navigation and clarifies expectations and assignments from the first accessing of a course (Morris and Finnegan, 2008; Vai and Sosulski, 2015). In addition, logical flow of content is conducive to course completion, important especially for voluntary training of noncompulsory courses (Morris and Finnegan, 2008). In student-centered learning, instructors also need to recognize that different learners have different learning preferences. These preferences - also addressed as “learning styles” - describe a learner’s preferred approach to gathering, processing, interpreting, and analyzing information. While evidence exists that the reading-writing preference varies between settings, text still prevails as the major information in online courses ( Kharb *et al.*, 2013). Thus, course designers should strive for an uncluttered and visually accessible layout.

Instructors should keep text clear and simple and strive for a uniform appearance between modules or learning units. Vai and Sosulski (2015) recommend an open layout, leaving white space to enhance readability and avoid overburdening the learner. Proper use of headings and subheadings guide the reader. It is recommended to organize the text into short paragraphs, insert meaningful breaks, and use bullet points if possible. Short lines work best on a computer screen and ensure readability independent of browser screens. Text should be left-justified with ragged right margins.

Web-safe typefaces are preferred and most likely preset in the LMS used for designing the course. If not, the use of sans serif (without serif) typefaces such as Arial or Helvetica is desirable due to the better readability. 12-point type for online is recommended. Additional general suggestions include avoiding all caps, and *italics.* Avoid using too many colors and strive for a clean aesthetic design. Use images sparingly and ensure that they relate to the text. Symbols and icons, on the other hand are effective signposts to important points (Vai and Sosulski, 2015).

## Tip 9: Provide Resources for Advanced Learning

While mandatory readings and multimedia should be uploaded in the course module, a module space for resources can be made available for additional reading. Oftentimes, a library site link is available through the LMS, but a short collection of readings curated by the expert provides additional perspectives of the field. If research projects are part of the assessment this is also the place for article collections. These collections can contain subfolders identifying that identify specific projects and fields. However, circling back to different learning preferences, it is advisable to offer multimedia resources besides textual material. A variety of presentation styles and images inspire students to find their own type of resources and go beyond the required materials to enrich a course topic (Vai and Sosulski, 2005).

## Tip 10: Build a Community of Learners Beyond the Course

Online learning provides ample opportunities for collaboration. Tools such as discussion forums are available through the LMS and allow for the exchange of perspectives and nurture a classroom community within the scope of a course (Mealy, 2015). Assignments such as team projects and presentations allow the instructor to additionally cultivate classroom collaboration and trust through a meaningful shared learning experience. However, with today’s multimedia online culture, communities of learners can be defined beyond the course and participate in a global community. Social media allows instructors to reach a broader community of learners. Technologies especially designed for a channel of communication and collaboration include a variety of experience- and resource-sharing tools that enable display of personal work along with reflective writings such as blogging ( Dabbagh and Kitsantas, 2012).

From the experience of the author, one great way to involve the global learning community is the creation of personal learning environments (PLEs). These are online web spaces created through free web hosting services or open-source content management systems, e.g., Weebly or WordPress, and allow presentation of assignments or reflective blogs to the public with the invitation for interaction. PLEs offer tools for attractive multimedia enrichment of educational work, such as the addition of images, YouTube videos, and podcasts. Combining an online course with adoption of PLEs not only inspires students to become effective self-regulated learners but also induces creativity and a sense of responsibility in connection with educational content. Finally, PLEs foster social interaction and learning communities through ease of access and a sense of belonging beyond the timeframe of a course. As an example of collaborative technology, Wikis are examples of a web-based tool for collaboratively editing content in real time using web browsers. Wiki websites allow users to contribute to projects regardless of geographic location, appealing to the community of online learners in healthcare education ( Rasmussen, Lewis and White, 2013; Vai and Sosulski, 2015).

## Tip 11: Faculty Development for Online Instruction

Designing online courses involves a systematic development of instructional materials and an analysis of the learning needs of a future audience, and the implementation of online structures to meet those needs. However, oftentimes, the course designer develops the course independently from faculty, and faculty development is needed to master the dynamic and complex relationship between content, pedagogy, and technology. The value of piloting a course has been described previously ( Lam *et al*., 2011; Mennin *et al.,* 2013; Mealy, 2019). Koehler and colleagues describe a transactional model of effective online teaching consisting of four elements: (1) content, (2) technology, (3) representation, and (4) pedagogy ( Koehler *et al*., 2004). The course designer can aid through explanatory videos that accompany the course.

Previous reports indicate successful instruction of online courses if the educator-designer and students collaboratively create the course in a design thinking approach ( Koehler *et al.,* 2004; Badwan *et al.,* 2018). However, even if the course was developed independently, there are several ways the educator can be supported in harnessing the expertise needed to successfully deliver an online course. First and foremost, an information technology instructor is needed who is familiar with the tools in the LMS and can deliver training sessions to faculty groups and individuals. Ideally, the information technology instructor is also available to troubleshoot with faculty and the course designer on short notice, if necessary. Centralized training ensures uniform course delivery and supports an institution-typical culture of delivery. Second, personalized coaching-based faculty development with the institutional faculty development expert allows for exploring the use of technology for desired learner-centered pedagogy for individual course activities. Last but not least, course facilitators must practice individual sessions with students or colleagues to gain feedback about clarity of instruction and the instructor’s online presence - and simply to allow the instructor to feel comfortable teaching an online course.

## Tip 12: Lessons Learned: Feedback About the Course

Both assessment and evaluation are the pillars of the instructor’s overall course effectiveness. Assessments of students are tied directly to the course outcomes, and assessments may include student presentations, PLEs, papers, tests, and discussion forums. In online courses, quality and quantity of interaction (through, for example, graded discussion boards) provide feedback about the relevance of the discussion for the learners. It must be noted that an engaged and caring teacher who maintains a strong presence - by providing ongoing feedback to students or engaging in discussion boards - will make a difference in the general interaction of learners. Overall, assessments reveal the degree to which the students have accomplished the learning outcomes. This is valuable feedback to consider for the course designer (Vai and Sosulski, 2015). The value of self-reflection for medical educators in gaining a deeper understanding of both self and the situation with the use of journaling or storytelling has been described previously ( Sandars, 2009).

The evaluation process allows stakeholders to make a decision about the course itself and is oftentimes provided by the institution. Course evaluation provides feedback and motivation for learners, educators, and course designers to continuously improve the course for the next iteration ( Thomas *et al*., 2016). This involves determining standards to assess the quality, collecting appropriate information, and then examining how the standards were met ( Vassar *et al.*, 2010).

Many program evaluation models exist, including objective-oriented approaches, management-oriented approaches, participant-oriented approaches, expertise-oriented approaches, and logic models ( Vassar *et al.*, 2010). In online courses, surveys can collect feedback such as learner satisfaction, active learning, teaching presence, flow and accessibility of content, study load, and other important predetermined parameters. Lessons learned perhaps from individual incidences, e.g., student feedback about a class text, need to be discussed and improved for the next iteration. If the educator was not the course designer, such feedback needs to be shared with the correct person in order to implement changes. Finally, feedback reports will be provided to stakeholders for future directions. This is also the time to provide outstanding examples of student work to showcase student accomplishments.

## Conclusion

Designing and implementing online courses for health professions education can be accomplished by using backward design for curricular components supported by online platforms to host the course (learning management system). Realizing familiar pedagogies of active learning through collaborative technology, creative use of collaborative web-based technology allows for the establishment of a community of learners. Prudent use of multimedia and social media piques interest and curiosity and invites diverse learners with different preferences to communicate and gain additional knowledge beyond the course objectives. Two-pronged faculty development that provides technical training and personal exploration of preferred active learning methods helps the faculty novice to successfully deliver online courses.

## Take Home Messages

• Designing online courses for health professions education can be accomplished using backward design guided by the learning management system.
• Active learning pedagogies can be productively employed using collaborative technology such as break-out rooms, white boards, and other means.
• Creative combinations of tools provided through the LMS with online use of collaborative technology allows faculty to establish a community of learners beyond the course.
• Use of multimedia and social media reach diverse learners with different learning preferences.
• Faculty development involving technical training and exploration of preferred active learning methods ensures successful delivery of online courses.

## Notes On Contributors

**Elisabeth Frieda Maria Schlegel**, PhD, MSc, MBA, MS (HPPL), Assistant Director, Faculty Development and Medical Education Research, Associate Professor, Science Education, Co-Director, Medical Students as Teacher Elective, Donald and Barbara Zucker School of Medicine at Hofstra/Northwell. ORCID ID: https://orcid.org/0000-0002-0279-1438

Dr. Elisabeth Schlegel is Associate Professor in the Department of Science Education at the Zucker School of Medicine at Hofstra/Northwell. As Assistant Director of Faculty Development and Medical Education Research she is responsible for a spectrum of institutional strategies including program development and innovative faculty development across the educational landscape from undergraduate to continuous medical education for the medical school as well the affiliated Healthcare system (Northwell Health) with a focus on coaching for faculty providing active learning to medical students.

## Declarations

The author has declared that there are no conflicts of interest.

## Ethics Statement

This research did not require Ethics Board approval because it does not involve human or animal subjects.

## External Funding

This article has not had any External Funding
