# Supplementary material for: Designing Online Courses: 12 Tips for Health Professions Educators
Source: MedEdPublish (2016). 2020 Jun 1;9:117. [Version 1] doi: 10.15694/mep.2020.000117.1 (PMC10702633; doi:10.15694/mep.2020.000117.1)
Supplement: Peer review history

# Reviewer report: Reviewer response for version 1

**DOI:** 10.21956/mep.20050.r30728

**Published:** 2020-06-12

**Authors:** Tharin Phenwan

**Affiliations:** 1 University of Dundee

This review has been migrated. The reviewer awarded 4 stars out of 5 This is extremely useful. Thank you for your work and I will definitely use these tips for my next module.I have no further comments from other reviewers but have one question, if I may.Do you have extra advice on Tip 7? Which one will be better between have one designated communication method i.e. email/discussion forum, or multiple methods of communication? On the latest module that I taught, I found that students prefer to use multiple methods of communication but some educators mention that they prefer it to be one method of communication.

Reviewer Expertise:

NA

No decision status is available

# Reviewer report: Reviewer response for version 1

**DOI:** 10.21956/mep.20050.r30727

**Published:** 2020-06-11

**Authors:** Hebat Allah A. Amin

**Affiliations:** 1 Department of Pathology, Helwan University, Faculty of Medicine

This review has been migrated. The reviewer awarded 4 stars out of 5 Thank you for this useful guide. It's well-structured and informative.I would like to suggest adding a checklist to facilitate the application of these wonderful tips. Your work is much appreciated.

Reviewer Expertise:

NA

No decision status is available

# Reviewer report: Reviewer response for version 1

**DOI:** 10.21956/mep.20050.r30730

**Published:** 2020-06-11

**Authors:** Nagwa Hegazy

**Affiliations:** 1 Menoufia university

This review has been migrated. The reviewer awarded 4 stars out of 5 concise informative article.Thanks for sharing.I had enjoyed the 12 steps description.

Reviewer Expertise:

NA

No decision status is available

# Reviewer report: Reviewer response for version 1

**DOI:** 10.21956/mep.20050.r30729

**Published:** 2020-06-10

**Authors:** Judy McKimm

**Affiliations:** 1 Swansea University

This review has been migrated. The reviewer awarded 5 stars out of 5 I really liked this 12 tips, there is a nice mix of the practical and theoretical which helps the educator design, plan and deliver meaningful online learning experiences whilst explaining why this is needed. Obviously this is particularly relevant right now during the pandemic but it is a useful article;e in its own right as well. I liked the mention of backwards design too because often we are stuck with our organisation's LMS and have to adapt to the constraints and opportunities within it. I would recommend this article for anyone just about to embark on programme design or conversion, as well as for those who are more experienced with online learning as it becomes the new normal.EDITORS - I just wanted to say that my view was all tangled up with the sections repeated or out of sequence

Reviewer Expertise:

NA

No decision status is available
